# Improvement of patient orientation and patient safety in health care – from competency-based and interprofessional medical education to cross-sector care

**DOI:** 10.3205/zma001240

**Published:** 2019-05-16

**Authors:** Jana Jünger, Eckhard Nagel

**Affiliations:** 1 Institut für medizinische und pharmazeutische Prüfungsfragen (IMPP), Mainz, Germany; 2 Universität Bayreuth, Rechts- und Wirtschaftswissenschaftliche Fakultät, Institut für Medizinmanagement und Gesundheitswissenschaften, Bayreuth, Germany

## Editorial

Patient orientation and patient safety play increasingly important roles within the debate about improving the quality of health care. At the conference of health ministers 2018, the aspect of informing and involving patients in the decision making process that affects their health was deemed to be a fundamental element of pioneering health policy. The national cancer plan (Nationaler Krebsplan, NKP) recommends to strengthen patient orientation as well [1].

This is in line with the “Masterplan Medizinstudium 2020”, which aims to empower healtcare professionals to focus on patients and their needs from the beginning [2]. This is primarily to be achieved by increasing interdisciplinary work processes and effective collaboration between health care professions. Therefore, it is essential to not only have common training and education programmes (measure <7>^1^), but also to implement it's practical realization into study courses and exams (inter alia, measures <6>, <14>, <15> and <23> up to <27>^2^). 

As a result, patient orientation and practical skills training will be revisited and defined as fundamental goals within the curricula, based on the framework of developing the German national competency-based learning objectives catalogue (Nationaler Kompetenzbasierter Lernzielkatalog Medizin, NKLM) and the redesigning and restructuring of faculty examinations, medical national licensing examinations and the corresponding examination learning objective catalogues (Gegenstandskatologe, GK). These topics are not only gaining importance in medicine but also in training programmes of the other health care professions. As an example, the Federal Union of German Pharmacistassociations supports this competency-based education in pharmaceutical studies in analogy to innovations in the medical studies [3]. So far, the existing learning objectives catalogue is hardly used in pharmaceutical studies and is not considered in the pharmaceutical examination learning objective catalogue and in national licensing examinations yet [3]. Furthermore, the upcoming reform of psychotherapy training will increase the importance of competency-based education as well. Health care professions like nursing, physiotherapy, speech therapy and midwifery are expanding their education programmes in line with international developments by further enhancing awareness for patient orientation and communication compentency [4]. This allows to strengthen the connection between the different health care professions even in education, and this contributes to increase the quality of patient care. 

This can also influence cross-sector care, which has been striving for some time as a central desideratum towards patient orientation in the health care system. In the last few years, a large number of laws has been passed which aim to improve patient orientation in care and cross-sector organisation of treatment guidelines [5], for example the law to optimize health care by social health insurance [6], the law to strengthen health promotion and prevention, discharge management of inpatients in accordance with Section 39 § 1A of the German Social Security Code V (SGB V) and the law for secure digital communication and applications in health care (“e-health law” – establishment of digital medical applications, e.g. electronic discharge reports, consistent medication plans, electronic patient files). In spite of this, the advisory council has concluded while evaluating of trends in health care system concludes that there is still a great segregation of in-patient and outpatient sector. This is due to poor opportunities for potential contract partners to change it [7].

How does this relate to education and examination? In particular, needs-based care is highly dependent on competencies of persons and professions acting within the existing structures. Due to changes in education of health care professionals it is possible to establish a central strategy that embraces an integrated and cross-sectoral care concept. To date, this field is hardly taken into account. Cross-sector, inter-professional, workplace-based training have not played a significant role in undergraduate and postgraduate education or national licensing examinations, excepting pilot projects, so far.

However, if we assume that early workplace socialization in established professional silo structures is formative for graduates and young professionals, we can already encourage openness to innovations and cross-sector improvements of the traditional professions. Current legal initiatives to reinforce patient orientation are perceived as a further work burden in education and labor-intensive day-to-day work routine. This perception is contradictory to the medical and health care professionals’ moral which is especially strongly felt by graduates and young professionals. It can be assumed that the current resignation and burnout rate of 20% in the student practical year and overworked state of junior doctors in their first year of employment are contributing factors. Burnout and overwork have been proven to lead to reduced patient safety and patient orientation [8], [9], [10]. 

Patient orientation also means a targeted strengthening of health literacy in everyday practice. Before consulting physicians many patients use online-sources to inform themselves [11]. To evaluate this information adequately, at least half of the patients in Germany would have to receive comprehensive training in their health literacy [12]. In addition, increasing digitization is changing the physician-patient relationship, which is very important for successful diagnostic and therapeutic planning [13].

The question is how to strengthen patient orientation and patient safety by making progress not only in education but also in faculty and national licensing examinations. Therefore, the integration of inter-professional and inter-sectoral topics in education as well as examinations of all health care professions is necessary. These topics should be based on competencies, be integrated in learning objective catalogues and previously content should be inter-professionally harmonized. To promote this the German National Institute for State Examinations in Medicine, Pharmacy and Psychotherapy has begun in January 2019 developing the competency-based learning objectives catalogues in pharmacy and psychotherapy simultaneously to the catalogue in medicine. The interdisciplinary and inter-professional fields include inter alia communication, intra- and inter-professional collaboration, professionalism, scientific competencies as well as leadership and management. The overarching focus is the inter-professional development of the learning objectives for patient safety and patient orientation.

With an aim to combine profession-specific and inter-professional education with cross-sector care a committee (GK-Commission) was founded. This committee is responsible for prioritizing topics at national level. Representatives of 50 health care institutions and organizations are involved in this GK-Commission: from health insurance companies to medical self-administration, from quality management to health policy, from patients to students. An overview of these institutions and organizations can be found at the following URL: [https://www.impp.de/informationen/kompetenzorientierte-gegenstandskataloge.html]. Based on supplied data the GK-commission reflects the direction and the specification in the graduate profiles and competences and gives impulses for new contents. This committee supports the development of the IMPP learning objectives catalogues for medicine, pharmacy and psychotherapy.

In conclusion, patient orientation and patient safety can be strengthened by the consistent further development of competency-based education and national licensing examinations, both profession-specifically and inter-professionally. Together with the encouragement of patient-side health literacy, this will make a substantial contribution to cross-sectoral and thus higher-quality health care.

## Notes

^1^<7>We expect universities, based on the experiences they have gathered, include an increasing number of common courses with students of other health care professions in their curricula. 

^2^<6> When reviewing the learning objective catalogues, the IMPP will reduce the number of examination topics and adapt them to the new learning targets, increasing the practical elements and focusing on general medicine. 

<14> In the future, clinical and theoretical content will be linked together from the first semester right up to the end of the studies. 

<15> Teaching medical practices shall be increasingly included in the medical education. Medical faculties will recruit new practices and qualify new teaching physicians to create a network of teaching medical practices. We expect medical associations, associations of statutory health physicians and medical professional associations to support this. The education itself shall remain under the auspices of the medical faculties. 

<23> After the preclinical part of the studies, a standardized national licensing examination is prescribed. This consists of a written part (after four semesters) and an oral-practical part (after six semesters). The oral-practical part will (where applicable) carried out as a structured clinical-practical examination which corresponds to the “Objective Structured Clinical Examination” (OSCE). 

<27> The IMPP develops standards for the introduction and implementation of the OSCE in the field of medical assessment. This also contains the standardization of the oral-practical assessment in general and the standards for the examiner´s qualification. 

## Competing interests

The authors declare that they have no competing interests. 

## References

[1] Bundesministerium für Gesundheit (2017). Nationaler Krebsplan. Aktueller Stand. Handlungsfelder, Ziele, Umsetzungsempfehlungen und Ergebnisse.

[2] Bundesminsterium für Bildung und Forschung (2017). Masterplan Medizinstudium 2020.

[3] Bundesapothekerkammer (2017). Kompetenzorientierter Lernzielkatalog Pharmazie – Perspektivpapier "Apotheke 2030". Empfehlungen der Bundesapothekerkammer. Verabschiedet von der Mitgliederversammlung der Bundesapothekerkammer am 29.11.2017.

[4] Küther G (2013). Die Akademisierung der therapeutischen Gesundheitsfachberufe in Deutschland. Eine Übersicht über bisherige Entwicklungen. Phys Rehab Kur Med.

[5] Jaeckel R, Schatz I, Amelung VE, Eble S, Hildebrandt H, Lägel R, Knieps F, Ozegowski S, Schlenker RU, Sjuts R (2015). Einfluss innovativer Versorgungsformen auf eine stärkere Patientenorientierung im Gesundheitswesen. Patientenorientierung: Schlüssel für mehr Qualität.

[6] Bundestag der Bundesrepublik Deutschland (2015). Gesetz zur Stärkung der Versorgung in der gesetzlichen Krankenversicherung (GKV-Versorgungsstärkungsgesetz) vom 16. Juli 2015. Bundesgesetzbl.

[7] Sachverständigenrat zur Begutachtung der Entwicklung im Gesundheitswesen (2018). Bedarfsgerechte Steuerung der Gesundheitsversorgung. Gutachten 2018.

[8] Koehl-Hackert N, Schultz JH, Nikendei C, Möltner A, Gedrose B, van den Bussche H, Jünger J (2012). Belastet in den Beruf - Empathie und Burnout bei Medizinstudierenden am Ende des Praktischen Jahres. Z Evid Fortbild Qual Gesundheitswes.

[9] Stefanescu MC, Sterz J, Hoefer SH, Ruesseler M (2018). Young surgeons' challenges at the start of their clinical residency: a semi-qualitative study. Innov Surg Sci.

[10] Panagioti M, Geraghty K, Johnson J, Zhou A, Panagopoulou E, Chew-Graham C, Peters D, Hodkinson A, Riley R, Esmail A (2018). Association between Physician Burnout and Patient Safety, Professionalism, and Patient Satisfaction: A Systematic Review and Meta-analysis. JAMA Intern Med.

[11] Bertelsmann Stiftung (2018). Wer suchet, der findet - Patienten mit Dr. Google zufrieden. Gesundheitsinfos Daten, Analysen, Perspektiven. Nr. 2.

[12] Schaeffer D, Vogt D, Berens E-M, Hurrelmann K (2016). Gesundheitskompetenz der Bevölkerung in Deutschland. Ergebnisbericht 2016.

[13] Bork U, Weitz J, Penter V (2018). Apps und Mobile Health - Viele Potenziale noch nicht ausgeschöpft. Dtsch Arztebl.

